# Survival Outcomes and Prognostic Factors in Patients with Meningioma: A Single-Center Study at the Indonesian National Cancer Center Dharmais Hospital (2019–2025)

**DOI:** 10.3390/curroncol33050237

**Published:** 2026-04-22

**Authors:** Rini Andriani, Sylvanie Ratna Permatasari, Ansi Rinjani, Mohammad Firdaus, Arwinder Singh, Oskar Ady Widarta, Rosalina, Achmad Fachri, Farilaila Rayhani, Nikrial Dewin, Aldithya Fakhri

**Affiliations:** 1 Department of Neuro Science, National Cancer Center Dharmais Hospital, Jakarta 11420, Indonesia; sylvanie@dharmais.co.id (S.R.P.); ansi.rinjani@dharmais.co.id (A.R.); muhammad.firdaus_spbs@dharmais.co.id (M.F.); arwinder@dharmais.co.id (A.S.); oskar@dharmais.co.id (O.A.W.); rosalina@dharmais.co.id (R.); achmadfachriradiologi@gmail.com (A.F.); farilaila.mdpath@dharmais.co.id (F.R.); nikrial.dewin@gmail.com (N.D.); 2 Faculty of Medicine, Universitas Indonesia, Jakarta 10430, Indonesia; aldithya95@gmail.com

**Keywords:** meningioma, overall survival, prognostic factors, extent of resection, adjuvant radiotherapy, WHO grade, Indonesia

## Abstract

**Background**: Meningioma is the most common primary intracranial tumor in adults, and survival outcomes are influenced by histopathological grade, tumor characteristics, and treatment strategies. This study aimed to evaluate overall survival (OS) and identify prognostic factors in patients with meningioma treated at a national referral cancer center in Indonesia. **Methods**: A retrospective cohort study was conducted at Dharmais National Cancer Center Hospital, including adult patients with histopathologically confirmed intracranial meningioma who underwent surgical resection between January 2019 and 17 August 2025. Overall survival was calculated from the date of histopathological diagnosis to death or last follow-up and analyzed using Kaplan–Meier methods and Cox proportional hazards regression. **Results**: A total of 114 patients were included (mean age 48.1 ± 10.5 years; 86.8% female), with most tumors classified as WHO Grade I (64.0%) and located at the skull base (57.0%). Subtotal resection was more common (67.5%), and 71.9% did not receive adjuvant radiotherapy. During follow-up, 14.0% of patients died, with cumulative overall survival rates of 95.6% at 6 months and 86.0% at 96 months. On multivariate analysis, only WHO tumor grade remained an independent prognostic factor (HR 2.199; 95% CI 1.161–4.167; *p* = 0.016), with higher grades associated with worse survival. Extent of resection and adjuvant radiotherapy were not significantly associated with overall survival after adjustment. **Conclusions**: In this Indonesian tertiary referral cohort, WHO tumor grade emerged as the only independent predictor of overall survival, underscoring its important prognostic role in meningioma; however, these findings should be interpreted with caution due to incomplete clinical data and relatively short follow-up duration. The high proportion of complex cases, including skull base tumors, reflects referral patterns and may also influence treatment outcomes.

## Simple Summary

Meningioma is the most common primary brain tumor in adults. While most cases are slow-growing with favorable outcomes, some show more aggressive behavior and reduced survival. Identifying prognostic factors is important to guide treatment and follow-up, particularly in resource-variable settings such as Indonesia. In this study, we analyzed survival outcomes of adult patients with meningioma treated at the Indonesian National Cancer Center Dharmais Hospital between 2019 and 2025. Most patients had low-grade tumors and favorable survival. However, higher tumor grade and incomplete resection were associated with poorer outcomes. Differences in survival among patients receiving radiotherapy likely reflect more advanced disease at presentation. These findings provide important real-world evidence from Indonesia to support better risk stratification, treatment planning, and follow-up strategies.

## 1. Introduction

Meningioma is the most common primary intracranial tumor in adults, accounting for approximately 39.3% of all central nervous system tumors, with 55.4% classified as benign [1]. These tumors arise from meningothelial (arachnoid cap) cells and are typically slow-growing, although a subset demonstrates more aggressive biological behavior with significant impact on morbidity and survival [2]. Despite generally favorable outcomes, survival varies widely depending on tumor grade, location, and treatment strategy, highlighting the importance of identifying prognostic factors.

In Indonesia, a lower-middle-income country with a population exceeding 273 million, CNS tumors represent a growing health burden. Primary brain and CNS tumors ranked 15th in incidence, with approximately 5964 new cases and 5298 deaths annually [3]. However, national-level epidemiological and survival data remain limited due to the absence of a comprehensive brain tumor registry. Most available evidence is derived from single-center studies. For example, a study at the National Brain Center Hospital in Jakarta reported 325 meningioma cases between 2020 and 2022, demonstrating an increasing trend in case detection [4].

However, the interpretation of these data must consider the structural limitations of the Indonesian healthcare system. Neurosurgical and neuro-oncological services remain unevenly distributed, with only approximately 371 neurosurgeons nationwide (around 1 per 725,000 population), most of whom are concentrated in major urban centers, particularly on Java [5]. Access to radiotherapy is similarly limited, with facilities available in only a portion of provinces and often associated with long waiting times [6]. In addition, Indonesia’s geographic characteristics as an archipelagic country pose significant barriers to healthcare access, as referral centers are primarily located in large cities, making timely diagnosis and treatment difficult for patients from remote areas. Socioeconomic factors, including relatively low per capita income and disparities in health insurance coverage, may further delay healthcare-seeking behavior and adherence to treatment. These limitations may lead to delays in referral and treatment, particularly for patients from rural areas [7]. Therefore, survival data from Dharmais National Cancer Center Hospital are particularly important, as this national referral center receives complex cases referred from various centers across Indonesia, providing an appropriate setting to evaluate their clinical characteristics.

According to the World Health Organization (WHO) classification, meningiomas are divided into Grades I, II, and III [8]. Most tumors are Grade I (80.1%), while Grade II accounts for 18.3% and Grade III for 1.5%, with recurrence rates increasing markedly from 7 to 23% in Grade I to 50–55% in Grade II and 72–78% in Grade III tumors [1,9]. Consequently, WHO grade remains the most important determinant of prognosis and long-term survival [10,11]. However, additional prognostic factors such as a high Ki-67 index, subtotal resection, progesterone receptor negativity, larger tumor size (≥3–5 cm), and non-skull-base tumor location may further worsen outcomes [12,13,14]. Although molecular biomarkers and radiomic models are increasingly used, WHO grade, together with clinical and radiological characteristics remains the cornerstone of prognostic factor analysis in patients with meningioma [15,16,17]. Given the need to evaluate survival and prognostic factors in complex cases, a retrospective cohort study at Dharmais National Cancer Center, as a national referral institution, is warranted. By analyzing meningioma cases diagnosed between 2019 and 2025, this study aims to identify prognostic factors associated with survival outcomes, thereby supporting more precise risk stratification and evidence-based therapeutic decision-making.

## 2. Materials and Methods

### 2.1. Study Design

This study employed a quantitative observational analytic design using a retrospective cohort approach. Secondary data were obtained from electronic medical records at Dharmais National Cancer Center. Collected variables included demographic characteristics, baseline clinical features, histopathological and immunohistochemical findings, pre- and postoperative magnetic resonance imaging (MRI) results, and treatment modalities. The study was conducted at Dharmais National Cancer Center following approval from the institutional ethics committee. Patient survival status (alive or deceased) was ascertained, with follow-up extending until 17 August 2025. Eligible cases were identified from patients treated between January 2019 and 17 August 2025.

### 2.2. Study Population and Sampling

The target population comprised all adult patients diagnosed with intracranial meningioma who received treatment and follow-up at Dharmais National Cancer Center during the study period. Study samples included patients who met the inclusion criteria, were not excluded based on predefined criteria, and had complete medical records. A purposive, institution-based sampling method was applied, and all eligible patients meeting the criteria were included, resulting in total sampling. The inclusion criteria were: (1) adult patients age >18 years; (2) availability of pre- and postoperative MRI; (3) histopathological confirmation of meningioma with or without immunohistochemical examination, including specimens obtained from external institutions that underwent histopathological review (re-expertise) at Dharmais National Cancer Center; and (4) patients undergoing treatment and follow-up at Dharmais National Cancer Center in 2019–2025. The exclusion criteria were incomplete medical records or inability to contact the patient for survival verification.

### 2.3. Variables and Data Analysis

Primary variables included demographic factors (age, sex), clinical presentation (seizures, headache, consciousness level, cranial nerve deficits, motor weakness, cognitive impairment), tumor characteristics (WHO grade, Ki-67 index, lesion number, tumor location), treatment-related factors (extent of resection, radiotherapy), and survival outcomes. Tumor grading was performed according to the WHO Classification of Central Nervous System Tumors (2016), which categorizes meningiomas into Grades I–III based on histopathological features associated with biological behavior and prognosis. In addition to grading, we also analyzed specific histopathological subtypes, including meningoepithelial, fibrous, transitional, anaplastic variants, etc., to further characterize tumor heterogeneity [8].

Extent of resection was determined using postoperative MRI performed more than two weeks after surgery and categorized as gross total resection or subtotal resection. Gross total resection (GTR) in meningioma refers to complete tumor removal with no visible residual disease, assessed by intraoperative evaluation and confirmed by postoperative contrast-enhanced MRI as the gold standard. In contrast, subtotal resection (STR) indicates incomplete removal, with residual tumor identified intraoperatively or on postoperative imaging [18]. All imaging findings were independently interpreted by several neuroradiologists as part of the standard diagnostic practice at our institution. However, in this study, the application of Simpson grading was limited due to incomplete postoperative data in many retrospective cases [19].

Adjuvant radiotherapy was categorized as absent (no radiotherapy received), incomplete (initiation without completion of the planned course), or complete (completion of the prescribed regimen). Radiotherapy was delivered according to national guidelines and individualized based on tumor characteristics and clinical condition. The primary technique used was three-dimensional conformal radiotherapy (3D-CRT). In general, treatment doses ranged from 45 to 60 Gy for lower-grade tumors and up to approximately 59.4–60 Gy for higher-grade tumors, delivered in standard fractionation [20].

Survival time was defined as the interval from the date of histopathological diagnosis to death or end of follow-up, measured in months. Data were tabulated and analyzed using SPSS (version 29.0), STATA (version 17), and EpiData (version 4.7) software. Descriptive statistics were presented as frequencies, percentages, or summary measures as appropriate. Survival outcomes were analyzed using survival analysis techniques, with multivariable analysis performed using Cox proportional hazards regression.

Given the relatively small number of events, this study may be underpowered for multivariable analysis, and the results should be interpreted with caution due to the potential risk of overfitting. Missing data were handled using a complete-case approach, with analyses restricted to patients with available data for each variable, and no imputation was performed.

### 2.4. Ethical Considerations

This study was conducted after obtaining approval from the Ethics Committee of Dharmais National Cancer Center. Patient confidentiality was strictly maintained, and all data were anonymized prior to analysis. The datasets generated and/or analyzed during the current study are available from the corresponding author upon reasonable request and with appropriate institutional approval.

## 3. Results

### 3.1. Population Characteristics

A total of 114 patients were included, with a mean age of 48.11 ± 10.48 years; most were female (86.8%) (Table 1). The majority were married (86.0%) and had completed senior high school (54.8%), while 68.4% were unemployed. The most common clinical manifestation was headache (62.3%), followed by cranial nerve palsy (35.1%), seizures (28.1%), and motor dysfunction (25.4%); decreased consciousness was rare (1.8%). Most tumors were WHO Grade I (64.0%), followed by Grade II (30.7%) and Grade III (5.3%). Ki-67 was not examined in 80.9% of cases. The majority of patients had solitary lesions (87.7%) and skull base location (57.0%). Subtotal resection was performed in 67.5% of cases, and 71.9% did not receive adjuvant radiotherapy.

### 3.2. Overall Survival and Follow-Up

**Figure 1:**
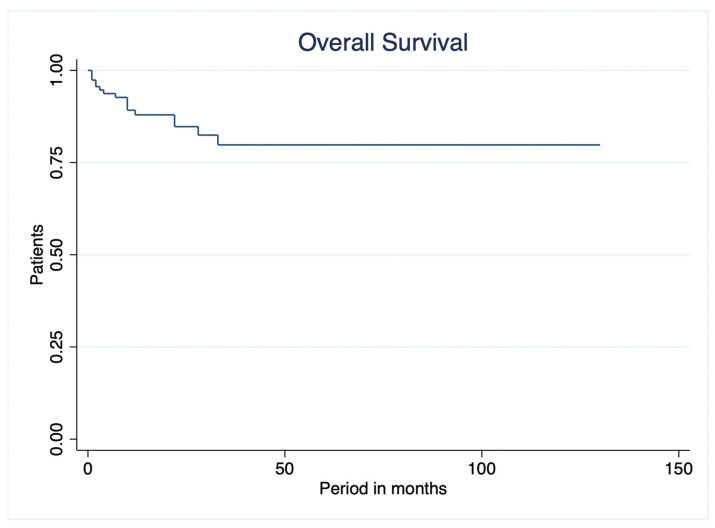
Kaplan–Meier overall survival curves for the entire cohort of meningioma patients. The curves show the percentage alive versus time after diagnosis of tumors based on histopathology in months.

During a mean survival period of 106.65 ± 5.55 months, 16 of 114 patients (14.0%) died. Overall survival was defined as the interval from histopathologically confirmed diagnosis to death from any cause or last follow-up. The cumulative overall survival rates were 95.6% at 6 months, 94.7% at 12 months, 93.9% at 24 months, 89.5% at 48 months, and 86.0% at 96 months. Because more than half of the cohort remained alive at the end of follow-up, the median overall survival was not reached (Figure 1).

### 3.3. Prognostic Factors Analysis

On univariate Cox proportional hazards analysis, age (HR = 0.961; 95% CI 0.927–0.995; *p* = 0.026) and WHO grading (HR = 2.233; 95% CI 1.158–4.306; *p* = 0.016) were significantly associated with overall survival. Adjuvant radiotherapy showed a trend toward significance (HR = 1.162; 95% CI 0.418–3.236; *p* = 0.083). Other variables, including gender, marital status, education, occupation, seizure, headache, decreased consciousness, cranial nerve palsy, motor dysfunction, histology type, Ki-67 index, number of lesions, lesion location, and extent of resection, were not significantly associated with survival. Variables with *p* < 0.05 in univariate analysis and those considered clinically relevant were subsequently included in the multivariate Cox proportional hazards model. In the multivariate analysis, only WHO grading remained an independent prognostic factor (HR = 2.199; 95% CI 1.161–4.167; *p* = 0.016). Age (HR = 0.974; 95% CI 0.934–1.016; *p* = 0.227), extent of resection (HR = 3.145; 95% CI 0.714–13.853; *p* = 0.791), and adjuvant radiotherapy (HR = 1.149; 95% CI 0.412–3.207; *p* = 0.130) were not independently associated with overall survival after adjustment (Table 2).

**Figure 2:**
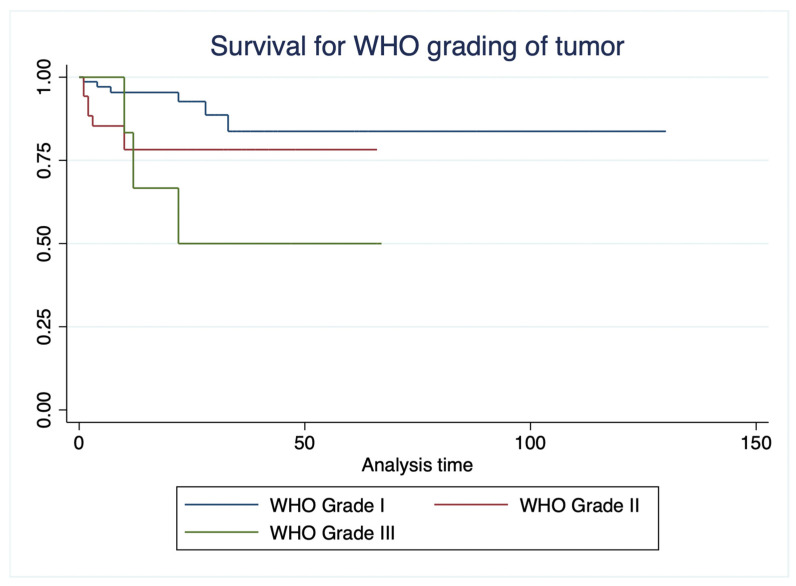
Kaplan–Meier survival curves for WHO grading of tumor.

Overall survival differed significantly across WHO tumor grades (log-rank χ^2^ = 6.368, df = 2, *p* = 0.041) (Figure 2). Patients with WHO grade I tumors demonstrated the most favorable outcomes, with a mean overall survival of 112.39 ± 7.19 months. In contrast, patients with WHO grade II and grade III tumors had progressively worse survival, with mean overall survival of 52.61 ± 4.50 months and 40.83 ± 10.78 months, respectively. These findings indicate a clear stepwise decline in survival with increasing WHO tumor grade, supporting its role as a significant prognostic factor.

**Figure 3:**
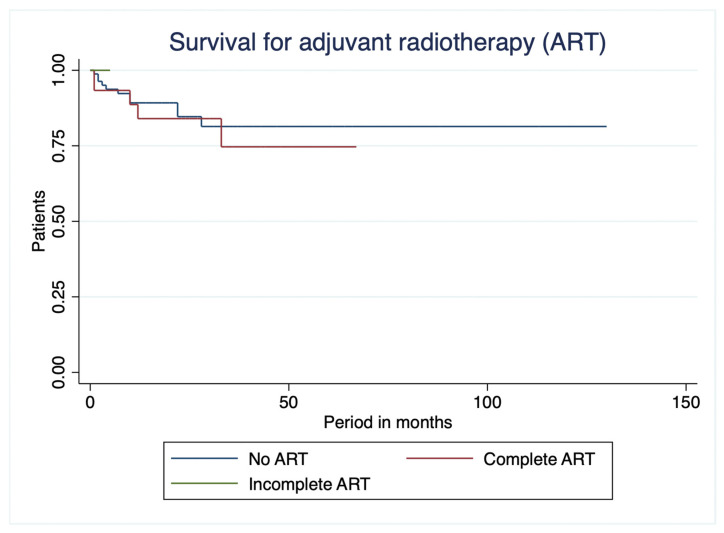
Kaplan–Meier survival curves for adjuvant radiotherapy (ART).

When patients were stratified according to adjuvant radiotherapy status, no significant difference in overall survival was observed between the groups (log-rank χ^2^ = 0.153, df = 1, *p* = 0.696) (Figure 3). Two patients who did not complete adjuvant radiotherapy were excluded from the analysis due to potential confounding effects. Patients who did not receive adjuvant radiotherapy demonstrated a longer mean overall survival (108.28 ± 6.23 months) compared to those who completed radiotherapy (54.20 ± 5.13 months); however, this difference was not statistically significant.

**Figure 4:**
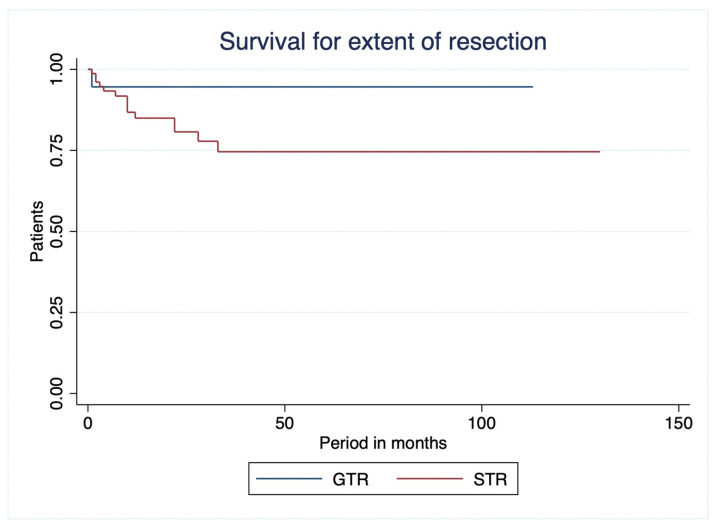
Kaplan–Meier survival curves for extent of resection. GTR: gross total resection, STR: subtotal resection.

Similarly, overall survival did not differ significantly according to the extent of tumor resection (log-rank χ^2^ = 2.433, df = 1, *p* = 0.119) (Figure 4). Patients who underwent gross total resection (GTR) had a mean overall survival of 106.94 ± 4.16 months, whereas those who underwent subtotal resection (STR) had a mean overall survival of 100.75 ± 7.06 months. Although survival appeared longer in the GTR group, the difference did not reach statistical significance.

### 3.4. Subgroup Analysis According to WHO Grading

Subgroup analyses were exploratory in nature and should be interpreted as hypothesis-generating rather than confirmatory. Subgroup analysis according to WHO tumor grading demonstrated variable effects of adjuvant radiotherapy on overall survival. In patients with WHO grade I tumors, those who did not receive adjuvant radiotherapy had significantly longer mean overall survival compared to those who completed radiotherapy (114.19 ± 7.90 vs. 39.92 ± 2.73 months; *p* = 0.049). A similar pattern was observed in WHO grade II tumors, where patients without radiotherapy exhibited better survival than those receiving radiotherapy (52.30 ± 5.42 vs. 37.97 ± 6.32 months; *p* = 0.005). In contrast, no significant difference was observed in WHO grade III tumors (41.00 ± 12.67 vs. 39.50 ± 19.44 months; *p* = 0.377). In the overall cohort, patients who did not receive adjuvant radiotherapy had significantly longer mean survival compared to those who completed radiotherapy (109.28 ± 6.23 vs. 54.20 ± 5.13 months; *p* < 0.001) (Table 3).

Subgroup analysis based on the extent of tumor resection across WHO grades did not demonstrate statistically significant differences in overall survival. In WHO grade I tumors, patients who underwent subtotal resection (STR) showed a longer mean overall survival compared to those who underwent gross total resection (GTR) (106.76 ± 8.76 vs. 73.15 ± 34.83 months; *p* = 0.391), although this difference was not statistically significant. Similarly, in WHO grade II tumors, mean survival was comparable between the GTR and STR groups (18.75 ± 11.65 vs. 20.00 ± 18.33 months; *p* = 0.106). In WHO grade III tumors, analysis was limited due to the small sample size, and no meaningful comparison could be performed. In the overall cohort, there was no significant difference in survival between patients undergoing GTR and STR (92.73 ± 11.18 vs. 101.63 ± 7.09 months; *p* = 0.372) (Table 4).

## 4. Discussion

In this study, the overall survival (OS) of meningioma patients treated at the Indonesian National Cancer Center Dharmais Hospital demonstrated generally favorable outcomes. The median OS was not reached, reflecting that the majority of patients remained alive throughout the follow-up period. The relatively high survival rates observed in this cohort may be explained by the predominance of low-grade tumors and the relatively young mean age of patients, although long-term outcomes may differ due to variations in access to adjuvant therapy and follow-up systems. Early mortality was low, and survival declined progressively over time, particularly among higher-grade tumors. The proportion of skull base meningiomas in our cohort appears higher than commonly reported, where convexity locations are typically more frequent [21]. This likely reflects referral bias, as our institution is a national cancer referral center that receives a higher proportion of complex cases, including skull base tumors, from secondary and regional hospitals. Minor revisions in the reported percentages following methodological adjustments did not alter the overall interpretation of these findings.

These findings are different from other relevant data in Indonesia. In Makassar, South Sulawesi, a study involving 65 patients over six years found a predominance of older (>47.5 years) female patients (78.5%), with the temporal region as the most common tumor location (30.8%) and headache as the leading symptom (31.1%). Notably, atypical meningioma was the most frequent subtype (29.2%), a higher proportion than typically reported [22]. In Bandung, West Java, 277 cases were reported between 2010 and 2013 (≈69 cases/year), with a subsequent 10-year study identifying 936 patients, reflecting a substantial and increasing burden. Most patients were female (88.0%) with a mean age of 44.16 ± 9.29 years, commonly presenting with visual disturbance (26.9%), headache (24.0%), and proptosis (23.5%). Sphenoorbital (23%) and convexity (21.7%) were the most frequent tumor locations [23]. In Jakarta, a study at the National Brain Center Hospital analyzing 325 patients (2020–2022) demonstrated a rising incidence trend, with a strong female predominance (4:1). Most tumors were WHO grade I, while higher-grade tumors occurred in slightly older patients. The most common locations were convexity (26.8%), falx (34%), and parasellar region (11.7%). Despite these inconsistencies, survival-specific data from Indonesian studies remain limited. The lack of comprehensive survival data, variability in histopathological reporting, and differences in treatment accessibility highlight important gaps in the current literature [4].

This study identified WHO tumor grading as the only independent prognostic factor for overall survival in multivariate analysis (HR 2.199; *p* = 0.016). A clear stepwise decline in survival was observed with increasing tumor grade, with mean OS decreasing from 112.39 months in grade I tumors to 40.83 months in grade III tumors. This finding is consistent with the current understanding that histopathological grade reflects tumor aggressiveness, proliferative activity, and recurrence potential. Durand et al. (2009) demonstrated that 5- and 10-year overall survival rates were 78.4% and 53.3% for Grade II tumors, compared with only 44.0% and 14.2% for Grade III tumors [24]. Progression-free survival was even more strikingly reduced in Grade III disease, with a 5-year PFS of 8.4% and no patients progression-free at 10 years. Multivariate analysis confirmed histological grade as one of the independent predictors of survival. Higher-grade meningiomas are characterized by increased mitotic activity, brain invasion, necrosis, and chromosomal instability, reflecting a more aggressive biological phenotype. In the updated WHO 2021 classification, additional molecular alterations such as TERT promoter mutations and CDKN2A/B homozygous deletions further define tumors with high-risk behavior and poor prognosis. These adverse histopathologic and molecular features likely underlie the markedly worse survival and steep early mortality observed in our WHO Grade II and especially Grade III subgroups [25].

Interestingly, extent of resection and adjuvant radiotherapy were not significantly associated with survival. Although gross total resection (GTR) is traditionally considered a key prognostic factor, the lack of significance in this study may be explained by several factors, including the predominance of skull base tumors (57%), which are technically challenging and often limit complete resection. The absence of a survival benefit from adjuvant radiotherapy is also notable. Patients who received radiotherapy tended to have worse survival, although this was not statistically significant. This likely reflects selection bias, where patients with more aggressive or higher-grade tumors were more likely to receive radiotherapy. Subgroup analysis further supports this interpretation, as radiotherapy appeared associated with poorer survival in grade I and II tumors, which contradicts biological plausibility and suggests confounding by indication [26].

According to the Indonesian National Guideline for Brain Tumors, maximal safe surgical resection remains the mainstay of treatment for meningioma, with the primary goal of achieving gross total resection (GTR) without compromising neurological function (Level I, Recommendation B). When complete resection is not feasible, a strategy combining subtotal resection (STR) with adjuvant therapy is recommended to optimize progression-free survival while minimizing postoperative morbidity. Preoperative embolization may be considered in selected cases to reduce tumor vascularity, induce tumor necrosis, and facilitate safer resection (Level II, Recommendation B), although its use depends on institutional capability. Furthermore, for skull base meningiomas (such as sphenoorbital lesions), the guideline recommends bone reconstruction to prevent temporal muscle atrophy and poor cosmetic outcomes (Level III, Recommendation C), highlighting the importance of multidisciplinary surgical planning. In the present study, STR was more frequently performed (67.5%), likely due to the high proportion of skull base tumors (57%), which are often associated with complex anatomy and limited surgical accessibility [20]. The survival benefit of gross total resection (GTR) is biologically plausible, as complete cytoreduction reduces tumor burden, eliminates proliferative clones, and delays recurrence. In contrast, subtotal resection (STR) leaves residual disease that may progress more rapidly, particularly in higher-grade or biologically aggressive tumors. Similarly, Przybylowski et al. (2022) showed that residual tumor volume is an independent predictor of progression, with volumes >3 cm^3^ significantly associated with poorer progression-free survival, even in WHO grade I skull base meningiomas [27].

Adjuvant radiotherapy constitutes an important component of meningioma management in the guideline, particularly for residual, recurrent, or high-grade tumors. According to the Indonesian National Guideline for Brain Tumors, WHO grade I–II meningiomas may be treated with conformal fractionated radiotherapy, stereotactic radiotherapy (SRT), or stereotactic radiosurgery (SRS), either directed at residual gross tumor or the tumor bed with a margin of 1–2 cm. In contrast, WHO grade III meningiomas are treated more aggressively, similar to malignant tumors, with radiation targeting the tumor bed and surrounding tissue using wider margins of 2–3 cm (Level I, Recommendation A). Radiotherapy may also be indicated in inoperable cases or as adjuvant therapy following surgery to reduce recurrence risk. However, in this cohort, the majority of patients did not receive adjuvant radiotherapy (71.9%), and no significant survival benefit was observed [20]. Regarding radiotherapy, management in Indonesia generally follows national guidelines, with dosing and fractionation adjusted based on tumor grade and patient condition. For lower-grade tumors, radiotherapy is typically delivered at 45–60 Gy in standard fractions, while higher-grade tumors receive approximately 59.4–60 Gy, with modifications for large tumor volume or clinical status. Hypofractionated regimens may be considered in elderly patients or those with poor performance status (Level IB, Recommendation A) [20]. Fischer et al. (2022) reported that adjuvant radiotherapy is consistently recommended for WHO Grade III and incompletely resected Grade II meningiomas, while its use in Grade I and selected Grade II cases remains variable and institution-dependent, reflecting ongoing uncertainty in standardizing its indications [28]. Such discrepancies likely reflect resource limitations, case complexity, and healthcare accessibility challenges in Indonesia. Importantly, the present study demonstrates that WHO tumor grading remains the most significant prognostic factor for survival, reinforcing the guideline’s emphasis on histopathological classification while highlighting the need for improved implementation of multidisciplinary, guideline-based care [4].

Meningioma management in Indonesia faces several important limitations, as reflected in this study. The high rate of subtotal resection highlights challenges in achieving optimal surgical outcomes, particularly for skull base tumors, likely due to limited access to advanced neurosurgical techniques, intraoperative navigation, and neurophysiological monitoring. The low utilization of adjuvant radiotherapy (26.3%) further suggests barriers such as limited facility availability, long waiting times, financial constraints, and issues with patient adherence, all of which may affect treatment continuity. In addition, the absence of Ki-67 data in most cases (80.7%) indicates gaps in pathological assessment, potentially limiting prognostic stratification [29]. As a single-center retrospective study, these findings may not fully represent national conditions, given regional disparities in healthcare access. To address these challenges, priorities should include establishing a multicenter national registry, improving access to advanced surgical and radiotherapy services, and strengthening referral systems to ensure timely and guideline-based care.

Several limitations of this study should be acknowledged. The mean follow-up duration was relatively short for a typically slow-growing tumor such as meningioma, potentially underestimating long-term mortality and recurrence patterns. Progression- or recurrence-free survival data could not be reliably assessed due to limited long-term follow-up at our center. Many patients, particularly those referred from outside Java, face geographic and logistical barriers to continued follow-up at our tertiary hospital. Additionally, within the Indonesian national referral system, patients are often referred back to secondary or regional hospitals after completing treatment, resulting in incomplete longitudinal data on disease progression or recurrence in our records [30]. Sociodemographic factors may also contribute to this limitation, as a substantial proportion of patients were unemployed (68.4%) and had lower educational attainment (Diploma 3.5%, Bachelor’s degree 9.6%, postgraduate 1.7%), which may affect adherence to long-term follow-up.

The incompleteness of certain data, including Simpson resection grade, is largely attributable to the transition toward electronic medical records (EMR) in our center and many hospitals in Indonesia. Comprehensive electronic medical records were only reliably implemented at our center around 2018–2019, resulting in incomplete data for earlier periods; therefore, the study period was limited to 2019–2025. Consequently, some historical clinical and surgical details were not consistently documented. In addition, the classification of subtotal resection encompasses a wide spectrum (1–99%), potentially introducing heterogeneity in outcome interpretation; however, further stratification was not feasible due to limited operative detail in some cases.

The retrospective single-center design introduces inherent risks of selection bias and limits generalizability to broader populations. The relatively small number of death events (n = 16) may reduce the statistical power of multivariate analysis and contribute to wide confidence intervals. Potential treatment-selection bias may have influenced the observed association between adjuvant radiotherapy and survival, as patients receiving radiotherapy likely represented higher-risk disease at baseline. Future long-term, multicenter studies incorporating molecular profiling are warranted to validate and expand these findings, particularly within Indonesian or Southeast Asian populations. This study reflects a real-world, single-center Indonesian cohort and may not fully represent the national population; multicenter studies are needed for broader validation.

## 5. Conclusions

In conclusion, this study demonstrates that WHO tumor grade is the only independent prognostic factor for overall survival in meningioma patients treated at our tertiary referral center. The predominance of complex cases, particularly skull base tumors, reflects referral patterns within the Indonesian healthcare system and may influence treatment strategies and outcomes. Although extent of resection and adjuvant radiotherapy were not independently associated with survival, their roles should be interpreted within the context of case complexity and clinical selection. Although gross total resection (GTR) is a known prognostic factor, its lack of significance in this study may reflect the high proportion of technically challenging skull base tumors. Similarly, the absence of a survival benefit from adjuvant radiotherapy likely reflects selection bias, as it was more often given to patients with more aggressive disease. Subgroup findings further suggest confounding by indication. According to the Indonesian National Guideline, maximal safe resection remains the mainstay of treatment, with adjuvant radiotherapy recommended for residual, recurrent, or high-grade tumors. Future studies with larger, multicenter cohorts, longer follow-up duration, and prospective design are needed to better evaluate long-term outcomes and minimize bias, as well as to support the development of more standardized national treatment and follow-up protocols.

## Figures and Tables

**Table 1:** Population Characteristics (n = 114).

Age, mean + SD	47.84 ± 10.12 years
Gender	Male 15 (13.2%), Female 99 (86.8%)
Marital status	Married 98 (86.0%), Unmarried 10 (8.8%), Divorced 2 (1.8%), Widowed 4 (3.5%)
Education	No formal education 7 (6.1%), Elementary school 17 (14.8%), Junior high school 10 (9.6%), Senior high school 63 (54.8%), Diploma 4 (3.5%), Bachelor’s degree 11 (9.6%), Postgraduate (>S1) 2 (1.7%)
Occupation	Civil servant/Military/Police 5 (4.4%), Private employee 26 (22.8%), Laborer 5 (4.4%), Farmer/Entrepreneur 5 (4.4%), Unemployed 78 (68.4%)
Clinical manifestation	
Headache	71 (62.3%)
Cranial nerve palsy	40 (35.1%)
Seizure	32 (28.1%)
Motor dysfunction	29 (25.4%)
Decreased consciousness	2 (1.8%)
WHO grading	
Grade I	73 (64.0%)
Grade II	35 (30.7%)
Grade III	6 (5.3%)
Histology types	
Meningothelial meningioma	55 (48.2%)
Atypical meningioma	30 (26.3%)
Transitional meningioma	10 (8.8%)
Anaplastic meningioma	6 (5.3%)
Fibrous meningioma	4 (3.5%)
Choroid meningioma	3 (2.6%)
Clear cell meningioma	2 (1.8%)
Angiomatous meningioma	2 (1.8%)
Psammmomatous meningioma	2 (1.8%)
Ki-67	
Not examined	92 (80.7%),
≤4%	7 (6.1%)
>4%	15 (13.2%)
Number of lesions	
Multiple	14 (12.3%)
Solitary	100 (87.7%)
Lesion location	
Non-skull base	44 (38.6%)
Skull base	65 (57.0%)
Mixed	5 (4.4%)
Extent of Resection	
Gross total resection	37 (32.5%)
Subtotal resection	77 (67.5%)
Adjuvant radiotherapy	
Absent	82 (71.9%)
Incomplete	2 (1.8%)
Complete	30 (26.3%)

**Table 2:** Hazard ratios for prognostic factors in survival.

Variables	Univariate Analysis		Multivariate Analysis	
	HR (95% CI)	*p*-Value *	HR (95% CI)	*p*-Value **
Age	0.961 (0.927–0.995)	0.026	0.974 (0.934–1.016)	0.227
Gender	2.231 (0.294–16.913)	0.437		
Marital status	0.481 (0.091–2.548)	0.390		
Education	0.962 (0.671–1.381)	0.835		
Occupation	0.894 (0.568–1.727)	0.656		
Seizure	1.514 (0.546–1.464)	0.432		
Headache	0.816 (0.292–2.276)	0.697		
Decreased consciousness	2.311 (0.296–18.017)	0.424		
Cranial nerve palsy	1.432 (0.503–4.074)	0.501		
Motor dysfunction	1.758 (0.624–4.949)	0.286		
WHO grading	2.233 (1.158–4.306)	0.016	2.199 (1.161–4.167)	0.016
Histology types	0.853 (0.697–1.293)	0.853		
Ki-67	1.105 (0.569–2.145)	0.768		
Number of lesions	1.988 (0.261–5.147)	0.326		
Lesion location	0.883 (0.348–2.243)	0.794		
Extent of resection	3.050 (0.692–13.440)	0.141		
Adjuvant radiotherapy	1.162 (0.418–3.236)	0.083	1.149 (0.412–3.207)	0.130

HR hazard ratio, CI confidence interval, * analysis using univariate Cox regression, ** analysis using multivariate Cox regression.

**Table 3:** Subgroup analysis of adjuvant radiotherapy according to WHO grading.

	Adjuvant Radiotherapy	Total Subject	Mean Survival (Months)	*p*-Value for Adjuvant Radiotherapy Impact (Log-Rank)
WHO grade I	Absent	55	114.19 ± 7.90	0.049
	Complete Overall	17 72	39.92 ± 2.73 112.34 ±7.19	
WHO grade II	Absent	22	52.30 ± 5.42	
	Complete Overall	11 34	37.97 ± 6.32 52.51 ± 4.52	0.005
WHO grade III	Absent	4	41.00 ± 12.67	0.377
	Complete Overall	2 6	39.50 ± 19.44 40.83 ± 10.78	
The entire cohort	Absent	82	109.28 ± 6.23	<0.001
	Complete Overall	30 112	54.20 ± 5.13 106.56 ± 5.56	

**Table 4:** Subgroup analysis of the extent of resection according to WHO grading.

	Extent of Resection	Total Subject	Mean Survival (Months)	*p*-Value for Adjuvant Radiotherapy Impact (Log-Rank)
WHO grade I	GTR	22	73.15 ± 34.83	0.391
	STR Overall	50 73	106.76 ± 8.76 102.11 ± 8.48	
WHO grade II	GTR	13	18.75 ± 11.65	0.106
	STR Overall	22 35	20.00 ± 18.33 19.56 ± 16.21	
WHO grade III	GTR	1	N/A	N/A
	STR Overall	5 6	35.4 ± 28.6 35.4 ± 28.6	
The entire cohort	Absent	37	92.73 ± 11.18	0.372
	Complete Overall	77 114	101.63 ± 7.09 103.46 ± 6.13	

N/A not applicable; GTR gross total resection; STR subtotal resection.

## Data Availability

The datasets generated and/or analyzed during the current study are not publicly available due to institutional data protection policies and patient confidentiality regulations but are available from the corresponding author upon reasonable request and with permission from Dharmais National Cancer Center.

## Abbreviations

The following abbreviations are used in this manuscript:

GTR	Gross total resection
STR	Subtotal resection
ART	Adjuvant radiotherapy
WHO	World Health Organization
